# Incidence and prevalence of traumatic and non-traumatic wounds and burns and access to wound care in Sierra Leone; data from a nationwide household survey (PRESSCO) 2020

**DOI:** 10.1016/j.heliyon.2024.e38693

**Published:** 2024-09-30

**Authors:** Jonathan H. Vas Nunes, Alex J. van Duinen, Daniel Boateng, Amidu J. Tommy, Osman Sankoh, Martin P. Grobusch, Håkon A. Bolkan, for the PRESSCO 2020 study group

**Affiliations:** a Department of Surgery, Amsterdam University Medical Centres, location AMC, Amsterdam, the Netherlands; b Global Surgery Amsterdam, Amsterdam, the Netherlands; c Masanga Medical Research Unit, Masanga, Sierra Leone; d Center of Tropical Medicine and Travel Medicine, Department of Infectious Diseases, Amsterdam University Medical Centres, location AMC, Amsterdam Infection and Immunity, Amsterdam Public Health, Amsterdam, the Netherlands; e Department of Surgery, Albert Schweitzer Hospital, Dordrecht, the Netherlands; f Clinic of Surgery, St. Olavs Hospital HF, Trondheim University Hospital, Trondheim, Norway; g CapaCare, Norway, the Netherlands, Sierra Leone; h Department of Public Health and Nursing, Norwegian University of Science and Technology (NTNU), Trondheim, Norway; i Surgical Department, ELWA Hospital, Monrovia, Liberia; j Department of Global Public Health & Bioethics, Julius Center for Health Sciences and Primary Care, University Medical Center Utrecht, the Netherlands; k Department of Epidemiology and Biostatistics, School of Public Health, Kwame Nkrumah University of Science and Technology, Kumasi, Ghana; l Deputy Vice Chancellor (Adm), University of Management and Technology (UNIMTECH), Kissy Dockyard, Freetown, Sierra Leone; m Honorary Professor, School of Public Health, Faculty of Health Sciences, University of the Witwatersrand, Johannesburg, South Africa; n Visiting Scientist, Heidelberg Institute of Global Health, University of Heidelberg Medical School, Heidelberg, Germany; o Adjunct Professor, Njala University, School of Community Health Sciences, Bo Campus, Bo, Sierra Leone; p Member, International Advisory Board, The Lancet Global Health, Sierra Leone; q Centre of Tropical Medicine and Travel Medicine, Amsterdam University Medical Centres, location AMC, University of Amsterdam, Amsterdam, the Netherlands; r Institute of Tropical Medicine, University of Tübingen, Tübingen, Germany; s Centre de Recherches Médicales en Lambaréné (CERMEL), Lambaréné, Gabon; t Institute of Infectious Diseases and Molecular Medicine (IDM), University of Cape Town, Cape Town, South Africa

**Keywords:** Sierra Leone, Wounds and injuries, Burns, Health care surveys, Accidents, Traffic, Neglected diseases, Needs assessment, General surgery, Healthcare inequalities, Help-seeking behaviour

## Abstract

**Objectives:**

This wound section of the PREvalence Study on Surgical COnditions (PRESSCO) determines the incidence and prevalence of wounds and burns in Sierra Leone. It further describes access to wound care and wound-related healthcare-seeking behaviour.

**Methods:**

Between October 2019 and March 2020, a nationwide cross-sectional household survey was performed. The survey was based on Surgeons OverSeas Assessment of Surgical Need (SOSAS). Additional questions relating to wounds and burns were added. Following randomization, 25 households in 75 clusters were sampled. Severe wounds were clinically examined.

**Results:**

Of the 3600 individuals included, 143 had developed 151 wounds, including burns (15.2 %) during the year preceding the interview (incidence 4.2 %). A total of 77 people had 83 wounds and burns at the time of the survey (prevalence 2.3 %), of which 23 were severe (prevalence 0.6 %). Burn incidence and prevalence were 0.6 % and 0.1 %, respectively. Most wounds were on extremities (73.5 %), often resulting from cuts (32.8 %), falls (22.4 %), or road traffic accidents (RTA; 16.4 %). Risk factors for developing a wound were male sex (p = 0.004), older age (p = 0.037) and smoking (p = 0.001). Severe wounds had a median duration of 18 months. For 70.2 % of wounds, care at a health facility was sought. Only 49.9 % of households reported financial capacity to visit a secondary health facility. For 56 (37.1 %) of wounds, the desired care was not obtainable. An estimated 44,000 (95 % CI 29,760–67,410) people in Sierra Leone suffer from a severe wound. An estimated 11,000 (95 % CI 6,416–18,268) annual deaths occur due to wounds, predominantly due to RTA's (66.7 %) and accidental injuries (20.0 %).

**Conclusions:**

Wounds and burns account for an extensive burden on the health and economics of the individual, the household, and the Sierra Leonean society. For over one-third of wounds, the desired surgical care was not obtainable.

## Highlights

• An estimated 40,000 people suffer from a severe wound in Sierra Leone.
• This includes burns, constituting 15.2 % of wounds.
• Only 49.9 % of households report financial capacity to visit a secondary health facility.
• Road traffic accidents account for 16.4 % of wounds and 66.7 % of wound-related deaths.
• There is a large unmet need for surgical wound- and burn care, including skin grafting.

## 1 Introduction

Severe wounds, including burns, cause considerable morbidity, mortality and economic burden globally - especially amongst those living in low-resource settings [1]. Although wounds remain broadly neglected and under-treated, there is a recent increase in advocacy to improve early diagnosis and treatment of pathogens causing wounds [[2], [3], [4]]. The major burden of disease caused by burns and its cost-effective surgical treatment, have led to classifying wounds as an essential surgical condition requiring global attention [5,6].

Sierra Leone has suffered from a civil war (1991–2002) and the West African Ebola outbreak (2014–2016). The country ranks 181 of 191 on the Human Development Index, and the health system is fragile [7]. More than 90 % of the surgical need in 2012 was unmet [8]. Many people in Sierra Leone have a poor nutritional status; for example, 30 % of the children under five years are stunted [9]. Therefore, they are at risk of disturbed wound healing. Data on prevalence, morbidity, and aetiology of wounds and burns from low-resource countries such as Sierra Leone are scarce [10]. In Rwanda, a prevalence of injuries and wounds grouped together was reported of 5.3 % [11]. Twenty-one percent of the household deaths in 2012 in Sierra Leone were reported to be caused by wounds (17 % injury-related and 4 % non-injury related) [12]. The same countrywide survey indicated that 25 % of the population had a surgical condition in need of attention; many of which due to wounds and burns [13]. These findings warranted further investigation as no physical examination was performed, and no information on health-seeking behaviour related to wounds and wound care was captured. The potential impact of empirically contributing factors - such as delay of presentation to a health facility, widespread usage of traditional medicine, lack of access to care and a high unmet need for surgical treatment such as wound debridement and skin grafting – remains unclear. Integrated Disease Surveillance and Response data from health centres reporting to the Ministry of Health and Sanitation (MoHS) is not publicly available and limited to numbers of patients diagnosed with a burn, trauma, tumour or skin infection and no validation details or follow up are available. Also unrecorded is the proportion of people with a wound or burn who receive the proper healthcare needed.

To the best of our knowledge, only a handful of health facilities in Sierra Leone have the capacity to perform specialized surgical wound care such as a skin graft, external fixation for fractures, osteomyelitis treatment, or amputation. No dedicated burn centres or nationwide burn trainings exist. Attention for burn care did increase and several trainings were provided after a tragic oil tanker accident in 2021 [14,15]. There is no official (inter-) national referral system for burn survivors.

The primary aim of this study is to establish the incidence and prevalence of wounds in Sierra Leone. Secondary aims are to identify risk factors, evaluate aetiology, wound characteristics and the impact wounds have on individuals and on mortality, and lastly to assess healthcare-seeking behaviour for wound care.

## 2 Methods

### 2.1 Study design

We conducted this PREvalence Study on Surgical Conditions (PRESSCO) in Sierra Leone from October 2019 until March 2020 (ISRCTN: 12353489). This was a nationwide cross-sectional household survey on the prevalence of several surgical conditions, including wounds and burns. For this study, 75 enumeration areas (EA) were sampled by Statistics Sierra Leone from 9671 nationwide clusters, based on a weighted random cluster design [16]. These clusters were based on the 2015 population-and-housing census in Sierra Leone [9]. Sampling was performed with a probability proportional to population size and stratified for distribution amongst the 16 districts of Sierra Leone and rurality. A map of the geographical distribution of the EAs and more methodological details have been described elsewhere [17]. All households were counted in the EAs and numbered, and 25 households were randomly selected by a random number calculator.

The PRESSCO survey was based on the SOSAS study, performed in Sierra Leone in 2012 [12]. Questionnaire-based interviews were held in two stages. First, all household heads were asked for informed consent and were asked general questions about the household, and a list of all members of the household, including the household head, was made. A household was defined as one or more persons who live together, share a meal, and slept in the same structure the night prior to the interview. For the second stage of the questionnaire, two household members were randomly selected from the household list and interviewed for surgical conditions. If household members were absent at the time of randomization, the household would be visited again, up to four times, before another household member would be interviewed. Enumerators were health care workers trained in surveying the PRESSCO 2020 questionnaire. Data was captured on tablets with an encrypted cloud-based Research Electronic Data Capture tool (REDCap) [18]. Exclusion criteria were limited to not providing informed consent.

### 2.2 Questionnaire

Questions about demography, socio-economic parameters, access to healthcare, general health and surgical history were asked. Additional questions relating to wounds and burns, healthcare seeking behaviour, the Wong-Baker FACES scale [19] and quality of life (Eq (5D) and VAS-score) [20] were added to the original SOSAS questionnaire from 2012, as supplemented by van Kesteren [17]. In addition to general questions on the household, the respective household heads were asked which household members died in the past 12 months, and what they considered to be the cause of death. The second stage of the questionnaire contained questions on demography, general health, medication and substance usage of the individual. All body parts were then verbally examined, blood pressure was measured and individuals with a wound were asked if they had visited a health facility for surgical consultation, and if not, why this was. In addition, they were clinically examined by a surgically trained enumerator. These enumerators were community health officers enrolled in a two-year surgical training program where advanced wound care was part of the training [21]. For quality control, photos were taken of severe wounds and assessed by a surgically trained medical doctor with an expertise in global health and tropical medicine. Informed consent for the photographs was obtained, and care was taken not to include identifiable body parts or tattoos. Wounds were dressed again afterwards. Data collection was checked and validated daily by a team or investigators on this study.

### 2.3 Data analysis

Images and data were uploaded to REDCap and verified daily for completeness by both a field supervisor and a member of the core research group. Password-protected laptops with SPSS 26.0, STATA 17.0 Standard Edition and Excel 16.50 were subsequently used for analysis of data and images. Descriptive analyses and two-tailed Fisher's exact- and two-sample t-tests were performed. Significance was determined at p < 0.05.

### 2.4 Definitions and classifications

A wound was defined as a break in continuity of bodily tissue, irrespective of periodicity and aetiology, and interchangeable with the word ‘ulcer’ [22]. We found no internationally accepted definition of wound severity. For this study, wounds are categorized based on wound size and duration, to differentiate between recent minor cuts, such as those occurring during daily chores, and severe wounds. Wounds clinically confirmed to be at least 5 cm in diameter or reported lasting longer than one month were classified as severe. All other wounds were categorized as non-severe. Three subgroups were further identified: (1) ‘wounds that started in the past year’; and/or (2) ‘wounds that were present at the time of the interview - now’; and lastly (3) ‘wounds present at the time of the interview and clinically confirmed to be severe’.

## 3 Results

### 3.1 Incidence and prevalence

A total of 3625 consenting individuals from 1854 households were interviewed. Twenty-five were excluded due to significant missing information from the interview. One person with a severe wound refused to have wound photos taken. The median age of all respondents was 20 years, the majority lived rurally (67.3 %) and was illiterate (67.5 %). *[*Table 1*: demography of study participants who developed a wound* *in* *the year preceding the interview and participants with a (severe) wound at the time of the interview]*

**Table 1:** Demography of study participants who developed a wound in the year preceding the interview and participants with a (severe) wound at the time of the interview.

	All		People with a wound developed last year		**P-value**	People with a wound now		**P-value**	People with a severe wound now^a^		**P-value**
*n* =	*3600*		*143*			*77*			*21*		
Sex											
Male	1699	47.2 %	87	5.1 %	0.004	49	2.9 %	0.010	15	0.9 %	0.040
Female	1899	52.8 %	56	2.9 %		28	1.5 %		6	0.3 %	
Missing	2	0.1 %	0			0			0		
Age											
0-4	513	14.3 %	10	1.9 %	0.037	5	1.0 %	0.000	1	0.2 %	0.001
5-14	927	25.8 %	32	3.5 %		7	0.8 %		0		
15-24	573	15.9 %	26	4.5 %		11	1.9 %		2	0.3 %	
25-46	1027	28.5 %	51	5.0 %		27	2.6 %		12	1.2 %	
> 46	560	15.6 %	24	4.3 %		27	4.8 %		6	1.1 %	
median (range. years)	20		25			20			20		
Residency											
Rural	2424	67.3 %	97	4.0 %	0.928	54	2.2 %	0.713	18	0.7 %	0.100
Urban	1176	32.7 %	46	3.9 %		23	2.0 %		3	0.3 %	
Education											
None	1091	30.3 %	43	3.9 %	0.495	30	2.7 %	0.622	9	0.8 %	0.438
Primary school	188	5.2 %	11	5.9 %		9	4.8 %		4	2.1 %	
Secondary school	561	15.6 %	29	5.2 %		18	3.2 %		6	1.1 %	
Tertiary	141	3.9 %	8	5.7 %		5	3.5 %		0		
Missing	8	0.2 %	0			0			0		
*Below 18 years old*	*1611*	*44.8 %*	*52*	*3.2 %*		*15*	0.9 %		*2*	0.1 %	

a A wound of at least 5 cm or existing more than 1 month.

**Fig. 1:**
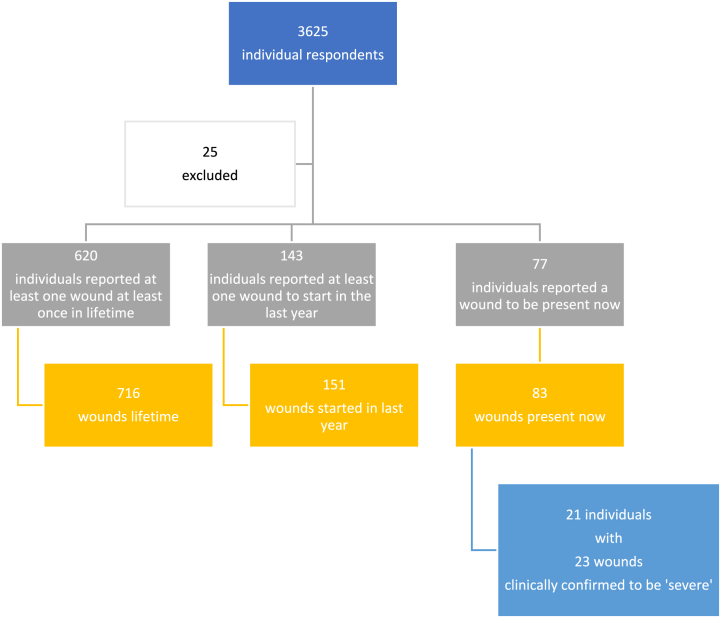
flowchart of respondents and wound numbers.

There were 143 individuals reporting a total of 151 wounds that started in the past 12 months, yielding an annual incidence of 3972 (95 % CI 3381–4662) per 100,000 population. Seventy-seven individuals reported a total of 83 wounds to be a problem now, yielding a prevalence of 2139 (95 % CI 1714–2666) per 100,000 population. Twenty-three of these wounds (in 21 individuals) were clinically confirmed to be severe, suggesting 583 (95 % CI 381–893) patients per 100.000 in Sierra Leone. Considering a population of 7,548,702 in Sierra Leone in 2021 [23], this equals 44,009 (95 % CI 28,760–67,410) *[*Fig. 1*: flowchart respondents and wound numbers].*

Twenty-three burns were reported by 21 individuals originating in the previous 12 months (incidence of 583 (95 % CI 381–893) per 100,000 people), leading to an annual 44,009 new burns in Sierra Leone.

### 3.2 Risk factors and characteristics of people with a wound

Wounds (including burns) that started in the past 12 months were significantly more often reported by males (p = 0.004) and people above 14 years old (p = 0.001; median age 25 years). No significant correlation was found between wounds and potential risk factors such as educational level, literacy, or residency.

People who reported a wound that started in the past 12 months reported a similar Eq (5D) score (5.96; 95 % ci 5.69–6.22) to that of the population (5.75; p = 0.126). They were more often cigarette smokers (17 % vs 9 %; p = 0.001). Only 3 % of the population had been tested for diabetes. No significant differences were found in alcohol consumption [Table 2: characteristics of people with a wound].

**Table 2:** Characteristics of people with a wound.

	All		People with a wound in last year		**P-value**
n =	3600		143		
Sickle cell (self) diagnosis					
Yes	66	1.8 %	2	3.0 %	0.187
No	3312	92.0 %	128	3.9 %	
Unknown	222	6.2 %	13	5.9 %	
Alcohol					
Yes	281	7.8 %	17	6.0 %	0.305
No	3265	90.7 %	125	3.8 %	
Unknown	39	1.1 %	1	2.6 %	
Smoking					
Yes	320	8.9 %	24	7.5 %	0.001∗
No	3242	90.1 %	117	3.6 %	
Unknown	28	0.8 %	0		
Diabetes (self) diagnosis					
Tested	110	3.1 %	5	4.5 %	
Positive	22	0.6 %	2	9.1 %	0.019
Negative	87	2.4 %	2	2.3 %	
Not tested	3208	89.1 %	118	3.7 %	
Unknown	282	7.8 %	20	7.1 %	
Eq (5d)(average^a^)	5.75		5.96		0.126

a p = 0.024 for above 17 years only.

Ten people reported a medical history that was directly wound related. Seven reported to suffer from *Wuchereria bancrofti* filariasis and three from leprosy. In addition, two responded that as a child, they had frequent bone pain or were tested positive for sickle cell anaemia.

### 3.3 Wound aetiology and distribution

**Fig. 2:**
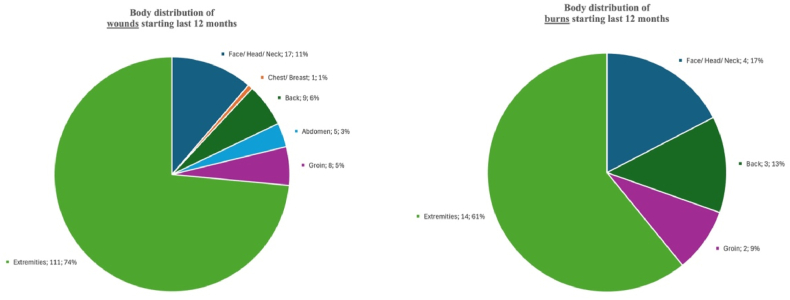
Distribution of wounds and burns.

Most wounds that developed, or were endured, during the past 12 months were on the extremities (n = 111; 73.5 %) and face, head or neck (n = 17; 11.3 %) [Fig. 2: distribution of wounds and burns]. Most were injury-related (n = 90; 59.6 %) or due to burns (n = 23; 15.2 %). For 116 wounds and 23 burns that started in the last 12 months, a trauma mechanism was reported. For wounds, a stab or cut was most common (32.8 %), followed by a fall (22.4 %) and road traffic accidents (RTA; 16.4 %). Burns were most often caused by a hot object or liquid (52.2 %), followed by open fire (13.0 %) [Table 3: aetiology of wounds and burns].

**Table 3:** Aetiology of wounds and burns.

	Total number of wounds or burns
Last 12 months	151
Wound not injury related	23 (15.2 %)
Wound injury related	90 (59.6 %)
Burn	23 (15 %)
(Recurrent) drainage/discharge	15 (10 %)
Wounds now	83
Wound not injury related	23 (27.7 %)
Wound injury related	54 (65.1 %)
Burn	4 (4.8 %)
(Recurrent) drainage/discharge	2 (2.4 %)

### 3.4 Characteristics of severe wounds

Twenty individuals had one severe wound, one person had three clinically confirmed severe wounds. The reported median duration of these wounds was 18 months, the median size was 4.8 cm. For twenty (87.0 %) of the severe wounds, respondents took oral analgesics at the time of questioning. For 60.0 % of the severe wounds, at least a non-steroidal anti-inflammatory drug (diclofenac, ibuprofen, naproxen or metamizole) was used. Two patients used additional traditional oral medicine. Sixty-one percent of wounds caused a pain VAS of four or higher, on a score of zero to ten. Twelve (52 %) of the severe wound(s) prevented the respondent from working at least sometimes. When considering all wounds present now, for 33 of the respondents (40.7 %), the wound hindered patients from working as before, and 14 (17.3 %) wounds resulted in the individual feeling ashamed *[*Table 4*: characteristics of severe wounds and its impact on pain, use of oral analgesia, and ability to work].*

**Table 4:** Characteristics of severe wounds and its impact on pain, use of oral analgesia, and ability to work.

	Severe wounds now	
*n* =	23	
Median duration of wound (months)	18	
Median size of wound (cm)	4,75	
Pain due to wound^a^		
0 (no pain)	0	
2 (hurts little bit)	7	30.4 %
4 (hurts little more)	6	26.1 %
6 (hurts even more)	5	21.7 %
8 (hurts whole lot)	3	13.0 %
10 (hurts worst)	0	
*No response*	2	8.7 %
Oral analgesia for this wound (days/week)		
Never	3	13.0 %
1 to 2	7	30.4 %
3 to 4	4	17.4 %
5 to 6	1	4.3 %
7 days a week	6	26.1 %
*No response*	2	8.7 %
Impact on work due to wound		
Still able to work always	7	30.4 %
Sometimes unable to work	8	34.8 %
Often unable to work	2	8.7 %
Unable to work	2	8.7 %
*No response*	4	17.4 %

a Wong-Baker FACES scale [19].

Seventeen of the individuals had wound photographs taken, one refused and for three patients, photographs were missing. For eleven of 17 (64.7 %) patients, the photograph suggested a continued need for surgical consultation. Three (27.3 %) patients reported to have undergone treatment at a hospital level.

### 3.5 Mortality

Of the 1854 included households, there were 354 deaths reported in the past 12 months by the household head. The household heads considered 15 (4.2 %) of these deaths to be due to a wound or burn: nine males and six females. There were thirteen adults and two children, with a median age of 32 years. Ten (66.7 %) deaths were due to an RTA, two due to a trauma with a hot liquid, and one due to a fall. With a mean size of the households of 5.2, the reported rate of deaths due to a wound or burn is 146 (95 % CI 85–242) per 100,000 population per year. This leads to an estimated 11,021 (6416–18,268) people dying from wounds every year in Sierra Leone.

### 3.6 Healthcare-seeking behaviour

Most heads of a household (79.8 %) responded having the (financial) means to allow for a visit to a primary health care facility; 49.9 % reported to have the means to visit a secondary health care facility. For 70.2 % of the wounds, a health facility was visited. For a total of 56 wounds (37.1 %), the desired healthcare was not obtained, with no significant difference in patient sex. Most often (39.3 %), this was due to insufficient funds. In 14 cases, a traditional healer, witch doctor, or bone setter was consulted, for a median duration of six weeks. Bone setters are traditional healers that specifically treat open or neglected fractures, often including severe wounds. Most wounds (25.8 %) were treated at a PHU (Peripheral Health Unit) or treated only with traditional medicine (5.3 %) or medication bought from the pharmacy (4.6 %). Five percent (5.3 %) of wounds were treated at a hospital *[*Table 5*: healthcare-seeking behaviour for wounds developed* *in* *the* *previous* *year and wounds present at the time of the interview].*

**Table 5:** Healthcare-seeking behaviour for wounds developed in the previous year and wounds present at the time of the interview.

	Wound in last year		Wound present now	
n =	151		83	
Medication for wounds currently (yes)	12	7.9 %	15	14.5 %
Visit to health facility for this wound (yes)	106	70.2 %	59	71.1 %
Reasons to not receive desired care for this wound				0.0 %
No time	4	7.1 %	1	2.9 %
Fear or no trust	2	3.6 %	1	2.9 %
Not available	2	3.6 %	1	2.9 %
No money for health care	22	39.3 %	19	54.3 %
No need (condition is not surgical)	18	32.1 %	4	11.4 %
Other. namely …	8	14.3 %	9	25.7 %
Total	56		35	
Visit to traditional healer. witch doctor. bone setter for this wound (yes)	14	9.3 %	21	25.3 %
Type of treatment (not wound specific question)				0.0 %
Minor procedures	87	57.6 %	32	38.6 %
Major procedure	6	4.0 %	7	8.4 %
None/No surgical care	13	7.9 %	18	21.7 %
Not reported	45	29.8 %	26	31.3 %
Type of wound care received				0.0 %
Traditional medicine oral	1	0.7 %	1	1.2 %
Traditional medicine topical	7	4.6 %	8	9.6 %
Modern medicine oral (bought from local pharmacy)	4	2.6 %	5	6.0 %
Modern medicine topical (bought from local pharmacy)	3	2.0 %	3	3.6 %
Dressing at peripheral health unit	39	25.8 %	5	6.0 %
Dressing at hospital	8	5.3 %	6	7.2 %
Other. namely …	6	4.0 %	9	10.8 %
Nothing	22	14.6 %	8	9.6 %
Not reported	61	40.4 %	38	45.8 %
Does the problem still impact your daily life?				0.0 %
The condition is not disabling	127	84.1 %	30	36.1 %
I'm not able to work like I used to	14	9.3 %	33	39.8 %
I feel ashamed	8	5.3 %	14	16.9 %
I need physical help with daily living	1	0.7 %	4	4.8 %
Not reported	1	0.7 %	2	2.4 %

Of the 83 wounds present now, 79 (95.2 %) were reported to need surgical care. Thirty-one (37.3 %) wounds did not receive the desired surgical attention. Seventy-six of all respondents (2.1 %) were taking medication related to a wound at the time of the interview.

For 18 burns, when asked for the first treatment sought, raw eggs (21.7 %), covering with mud (21.7 %), palm oil (8.7 %), Aloe Vera (8.7 %) salt (4.3 %), gentamycin violet (4.3 %), iodine cream bandages (4.3 %) or papaya fruit (4.3 %) was used. No skin graft was reported.

## 4 Discussion

### 4.1 Main findings in perspective

This study suggests that over 40,000 people in Sierra Leone suffer from a severe wound. An estimated 11,000 annual deaths occur due to wounds, two thirds from RTAs. A scarcity of data and lack of consensus on the definition of 'wound' make comparison of these numbers to historical data and to statistics of other countries challenging. A systematic review on prevalence on chronic wounds - over three weeks old - in 2019 yielded only 11 eligible studies from middle- and high-income countries, showing a prevalence of 221 per 100,000 population [10]. In addition, a study in Uganda in 2014 reported a prevalence of 2260 wounds in need of surgical consultation per 100,000 people, an outcome similar to this study [24]. As expected, severe wounds are more common in Sierra Leone than in middle- and high-income countries.

Living in rural areas, being illiterate, male, adult and smoking were associated with a higher likelihood of reporting a wound. Only one half of households reported to be able to pay for a visit to a secondary health facility. Financial constraint was the strongest barrier found in access to care, leading to over one third of wounds not receiving the desired surgical attention.

### 4.2 Burns

Challenges to burn care in Sierra Leone are suggested to exist on multiple levels. Although this study suggests that there is a lack of access to care, burn survivor numbers in this study are too small to identify whether this is due to challenges in infrastructure, finances, human resources or health care capacity or delivery. However, empirically, all these factors are likely to be at play. In terms of burn incidence numbers, a household study in Uganda in 2017 described a prevalence of burns in need of surgical consultation of 400 per 100,000 population, nearly four times as much as reported in this study [24]. Heterogenous definitions on burns complicate comparison to our data. In addition, studies on incidence of burns often describe high resource settings, hospital data, cumulative incidence numbers or burns amongst children [25].

### 4.3 Healthcare-seeking behaviour

Seventy-six people reported that they currently take medication for a wound. Over half of patients with a severe wound use a NSAID, known for e.g. nephrotoxicity and gastric side effects. Only nine percent of people with a wound in the year prior to the survey responded to have visited a traditional healer. This is lower than, for example, the 46 % of Ebola survivors in Sierra Leone who used traditional or complementary medicine [26]. For more than a third of wounds, surgical care was not obtainable. Only five percent of people received surgical care at a hospital level. The wound photos in this study underline that two thirds of people with a severe wound are still in need of surgical care and that roughly one third do not access proper wound care at a hospital level. With a median duration of severe wounds of 18 months, there is therefore a high untreated burden of disease. This may be increased by secondary infection of wounds and associated morbidity and mortality.

Most of the initial burn treatment was done with use of traditional materials such as raw eggs, mud, salt, papaya or palm oil. Interesting data exists on the efficacy of some of these treatments, such as egg-white, papaya and coconut oil [27,28]. They are in keep with data from Ghana and Ethiopia [29,30]. However, the fact that nearly all burns were treated with traditional materials, and since no one in this study reported having undergone a skin graft, may also point out a lack of (access) to surgical wound and burn care.

### 4.4 Possible explanations and implications for clinicians and policymakers

Initiating a healthcare insurance, providing in-hospital meals, or including people with severe wounds or burns in the free healthcare initiative [31] may potentially ameliorate the financial barrier to access to wound and burn care. Forty percent of people with a wound is unable to work like before and 17.3 % feels stigmatized, increasing the economic burden on society as a whole. Improving the surgical system in Sierra Leone and increasing availability of and access to quality wound and burn care are therefore important elements to meet multiple sustainable development goals [32]. These elements include the development of burn centres and improving human resources and training for burn care. They also include designing a regional or national burns referral system for acute burn survivors, but also for the late, and often chronic, sequalae such as contractures and deformities.

From an African and global perspective, although death rates due to injury are declining, injury rates and the associated economic burden are rising [[33], [34], [35]]. In fact, the Global Health Observatory shows that in Sierra Leone, both the injury rates, and death rates due to injury, are rising [36,37]. Trauma accounted for 11.6 % of attendances of the Emergency unit and 68 % of adult surgical admissions at Connaught Hospital, the largest hospital in Sierra Leone's capital Freetown, in 2019 [38]. Investment in the prevention of road traffic accidents and accidents with open fire, as well as improvements in trauma care could therefore play a role in future decisions to strengthen the health system in Sierra Leone [39].

### 4.5 Strengths and limitations of this study

SOSAS studies have been criticised for lack of physical examination and granular data on the specific surgical conditions surveyed [39]. A strength of this particular study design was the inclusion of physical examination by a surgically trained healthcare professional. This allowed for a more precise determination of prevalence of severe wounds and burns and was a major reason to repeat the SOSAS questionnaire. Another strength was the addition of wound photos and questions relating to wound care, quality of life and healthcare seeking behaviour, allowing for more granular data. A third strength was that recollection bias, albeit not ruled out, was reduced by performing analyses only on wounds that were reported to have started during the past 12 months, and/or those that were present during the survey.

Limitations of this study include firstly the lengthy questionnaire, requiring interviews of up to 1 h depending on how many conditions were reported. Secondly, a wound was only recorded and physically examined if it was reported by the respondent. Thirdly, no true definition of ‘wound’ or ‘severe wound’ exists, limiting comparison with other studies [40]. Fourthly, surgical conditions in this study could only be labelled once. Under-reporting of a burn or wound therefore cannot be excluded; e.g., when a condition is classified as a deformity due to a contracture, rather than as wound or burn. Fifthly, a selection bias of including less healthy males cannot be ruled out, e.g. males in the military are not included in the households. Sixthly, underreporting of the use of traditional or complementary medicine cannot be ruled out, e.g. due to shame of the respondent because of enumeration by surgically trained personnel. Seventhly, no direct questions on income and assets were included in the questionnaire. Therefore, only proxies for socio-economic wealth were used in this study. Data from the Demographic Health Survey in 2019 however suggest that proxies such as education have a strong correlation with wealth in Sierra Leone [9].

Both a strength and limitation of this study is the repetition of more, and more specific, questions from the SOSAS study in Sierra Leone in 2012. Data suggests a reduction in prevalent wounds in need of surgical attention from 109 per 1000 people in 2012 to 22 per 1000 in 2020 (reduction of 79.8 %). This would be further reduced to 6 per 1000 people, when considering only severe wounds. This reduction has limited the power of this study. The only significant differences in baseline characteristics of the two studies, were that the population in 2020 was more rural (66.9 vs 61.2 %, p < 0.001) and had smaller households (mean 5.2 vs 6.4 individuals, p < 0.001) [17]. No clear explanation for the reduction in surgical condition was found. Interpretation is limited due to differences of methodology between the two studies, such as the addition of physical examination and enumeration by surgically trained healthcare staff. A possible explanation may be relative underestimation in 2020 or overestimation of the surgical need in 2012. The latter was also suggested by van Kesteren when comparing the 25 % surgical need found in 2012 to prevalence numbers from Nepal (10 %) and Rwanda (12 %) [11,41]. Other potential explanations are a decreased surgical need for wounds, e.g. due to increased access to healthcare leading to earlier wound care, temporary influx of external healthcare funding during the West-African Ebola virus disease outbreak from 2014 to 2016, or an increased attention to surgical care in Sierra Leone by the 10.13039/100009647Ministry of Health and Sanitation and the initiation of a surgical task sharing programme by CapaCare in 2011 [21].

### 4.6 Future research

Future research is proposed in overcoming barriers to access to quality and timely wound care. These include cultural barriers, measures for monetary barriers such as universal healthcare coverage and surgical and wound care capacity building such as burn units and wound clinics with options for skin grafting. Research on nutrition and knowledge, attitude and practice regarding wounds and burns may improve knowledge on less obvious challenges for prevention of wounds and burns and barriers in obtaining proper care. No data on what portion of reported medication usage were antibiotics was collected. Considering the high antimicrobial resistance rate found in Sierra Leone amongst wound pathogens, further research on antibiotic usage and stewardship is already suggested [42]. In addition, further research on RTAs and burns and risk factors for wounds such as smoking and nutrition may provide tools for prevention strategies. Future data collection on surgical conditions is suggested to be implemented in the Demographic Health Survey of Sierra Leone.

## 5 Conclusion

This study suggests that an estimated 44,000 people in Sierra Leone suffer from a severe wound or burn. Over one-third of wounds do not receive the desired surgical attention, in nearly forty percent due to financial constraints. The median duration of a severe wound is 18 months, and an estimated 11,000 annual deaths occur due to wounds, predominantly from road traffic accidents. Thus, wounds and burns are a heavy burden on the health and economics of many in Sierra Leone.

## Ethics statement

This study was reviewed and approved by the (1) Masanga Medical Research Units Scientific Review Committee, (2) the Norwegian Regional Committee for Medical and Health Research Ethics and (3) the Sierra Leone Ethics and Scientific Review Committee with the respective approval numbers: (1) MMRI-SRC-009-2019, (2) REC 2019/31932 and (3) SLESRC 2019/October/03. Informed consent was obtained from district, village, and household leaders, as well as every individual participant or parent/guardian. Additional informed consent was obtained for wound photographs from patients with a severe wound.

## Funding

This study was funded by the 10.13039/100009123Norwegian University of Science and Technology (10.13039/100009123NTNU), CapaCare and the University of Amsterdam's Center of Tropical Medicine and Travel Medicine. In-kind contributions were made by Statistics Sierra Leone, Masanga Medical Research Unit and the authors of this study. These funding bodies' and individuals' contributions had neither influence on the methodology or outcome of this study, nor on the decision to pursue publication. Financial allowances were provided for Sierra Leonean enumerators, independent from their data collection.

## Data sharing

The data supporting the findings in this study are available withing this article. The original dataset may be requested by contacting the corresponding author.

## Declaration of competing interest

The authors declare that they have no known competing financial interests or personal relationships that could have appeared to influence the work reported in this paper.

## PRESSCO 2020 study group members

Amsterdam UMC, Department of Surgery, Amsterdam, The Netherlands (Jurre van Kesteren, Jonathan Vas Nunes); CapaCare, Trondheim, Norway (Håkon A. Bolkan, Alex J. van Duinen); CapaCare Netherlands, Wageningen, The Netherlands (Josien Westendorp); Centre of Tropical Medicine and Travel Medicine, Amsterdam University Medical Centres, University of Amsterdam, The Netherlands (Martin P. Grobusch, Hanna M. Mathéron, Giulia L.E. Mönnink, Jonathan Vas Nunes); Clinic of Surgery, St. Olavs Hospital HF, Trondheim University Hospital, Trondheim, Norway (Håkon A. Bolkan, Alex J. van Duinen); Global Surgery Amsterdam, Amsterdam, The Netherlands (Jurre van Kesteren, Jonathan Vas Nunes); Institute for Health & Equity and Epidemiology Division, Medical College of Wisconsin, Milwaukee, WI, USA (Laura D. Cassidy); Johns Hopkins School of Medicine, Baltimore, USA (Reinou S. Groen); Department of Public Health and Nursing, Norwegian University of Science and Technology (NTNU), Norwegian University of Science and Technology, Trondheim, Norway (Håkon A. Bolkan, Alex J. van Duinen, Sara K. Hoel, Sofie M. Løvdal, Mia N. Østensen, Helene Solberg, Josien Westendorp); Department of Public Health Department Bonaire (Daniel van Leerdam); Department of Global Public Health & Bioethics, Julius Centre for Health Sciences and Primary Care, University Medical Centre Utrecht, Utrecht University, The Netherlands (Daniel Boateng, Kerstin Klipstein-Grobusch); KIT, Royal Tropical Institute, Amsterdam, The Netherlands (Daniel van Leerdam); Masanga Medical Research Unit, Sierra Leone (Diede van Delft, Martin P. Grobusch, Hanna Mathéron, Jonathan Vas Nunes); Sierra Leone, Statistics Sierra Leone, Hill Tower, Freetown (Silleh Bah, Abubakarr Kamanda-Bongay, Sonnia-Magba Bu-Buakei Jabbi, James A. Medo); Sierra Leone, University of Management and Technology (UNIMTECH), Freetown (Osman Sankoh); SOSAS—Surgeons Over Seas, New York, USA (Reinou S. Groen); University Medical Centre Utrecht, Utrecht, The Netherlands (Daan van Herwaarden, Janine P.J. Martens); Vrije Universiteit, Faculty of Medicine, Amsterdam, The Netherlands (Karel C. Lindenbergh).
